# Evaluation of the caffeine effect in the vestibular test

**DOI:** 10.1016/S1808-8694(15)31245-3

**Published:** 2015-10-20

**Authors:** Lilian Felipe, Lilia Correia Simões, Denise Utsch Gonçalves, Patrícia Cotta Mancini

**Affiliations:** Speech therapist, Hospital das Clínicas, UFMG; Speech therapist, Hospital das Clínicas, UFMG; Ph.D. in Tropical Medicine, Medical School, UFMG. Otoneurologist, Joint Professor, Department of Ophthalmology, Otorhinolaryngology and Speech and Hearing Therapy, Medical School, UFMG; Master in Linguistics, FALE-UFMG, Speech Therapist, Assistant Professor, School of Speech and Hearing Therapy, UFMG. Federal University of Minas Gerais

**Keywords:** vestibular function tests, caffeine, drug effects

## Abstract

Exist controversy about the interference of the caffeine in the vestibular test. Coffee is the richest source of caffeine. While in some services, the patients were orient to suspend the ingestion of caffeine 24 to 48 hours before the vestibular test, other not consider the suspension of this drink necessary.

**Aim:**

To evaluate the effect of caffeine in the vestibular test result.

**Study design:**

clinical with transversal cohort.

**Material and Method:**

Seccional and matched research. The vestibular test was performed twice in the same patient, with five days interval between the exams. In the first test, the patient did not drink coffee 24 hours before the exam; in the second, the patient drunk coffee as usual. All of the participants had clinical indication for vestibular test and were used to drinking coffee.

**Results:**

Nineteen women, medium age of 49,5 years, participated. The average coffee consumption was three cups per day. The complaints of anxiety and headache were associated with the submission to the vestibular test without coffee. The exams were not statistically different comparing the results of the tests performed with and without the coffee ingestion.

**Conclusion:**

The moderate ingestion of coffee was not shown to interfere in the results of the vestibular test. Considering that it is recommended that the patient be calm to be submitted to the vestibular test and that the half-life of the caffeine is only of six hours, we suggest that the orientation of complete and abrupt drinking coffee suspension of moderate dose before the vestibular test for the individuals used to daily drinking coffee be reevaluated.

## INTRODUCTION

Caffeine is the most used psychoactive substance in the world^1^. About 80% of the general population uses this substance daily, be it through coffee, tea, chocolate, soft drinks or caffeine-based drugs. Coffee is the richest source of caffeine^2^. After oral intake, caffeine is quickly eliminated with a half-life of four to six hours^3^.

The moderate intake of coffee is defined as daily intake that ranges from 200 to 300 milligrams^2^. Excessive intake is defined above 600 mg/day^4^. One cup of Brazilian coffee (60ml) normally has 50.4 mg caffeine^2^, and according to the authors it may vary from 85 mg to 125 mg^5^.

Under experimental conditions, caffeine, in moderate doses, produces excellent physical and intellectual performance, increase in concentration and reduction in time of response to sensorial stimuli^6^. Conversely, high doses may cause perceptible signs of mental confusion and induction of mistakes in intellectual tasks, anxiety, nervousness, muscle tremors, tachycardia and tinnitus^3^^,^^6^. Sudden interruption of caffeine intake may lead to withdrawal syndrome. The most common symptoms are fatigue, anxiety and depression, nausea, vomiting, headache and reduced concentration^7^^,^^8^. The occurrence of anxiety with sudden suspension of caffeine may be present even in subjects that take low doses of the substance (50 to 150 mg/day)^9^.

Instructions about coffee intake interruption before vestibular tests vary from service to service. Some authors recommend that the patients interrupt coffee intake 72 hours before the test^10^^,^^11^. Others indicate interruption for 48 hours^12^^,^^13^ or for 24 hours^14^^,^^15^. There are those that recommend no intake on the exam day^16^^,^^17^ and other that make no restrictions to intake18., 19., 20..

It is essential to maintain the patient calm during the performance of the vestibular test because stress and anxiety may lead to changes in tracing, especially caloric hyperreflexia^21^^,^^22^, inducing the mistaken diagnosis of vestibular disease^18^. The present study aims at assessing the effect of moderate doses of coffee in the vestibular test.

## METHODS

The present study was a comparative, transversal, matched analysis in which the studied group comprised patients that had taken caffeine, according to their own habits, and the control group was the same patients without the use of the substance. Considering that among the foods that contain caffeine coffee is the one that contributes the most to its intake^2^, researchers defined the concentration of caffeine based on daily coffee intake of participants.

The study comprised subjects sent from different primary care centers in Belo Horizonte with otorhinolaryngologists' referral to perform vestibular test.

Nineteen patients with vestibular symptoms that used to drink coffee everyday were enrolled in the study. None of them had been previously submitted to the test or had clinical presentation compatible with central labyrinth pathology.

First, patients were submitted to vestibular test following the instruction provided by the Service of Audiology, Hospital das Clínicas, Medical School, UFMG, which were: interruption of non-essential drugs and alcoholic drinks for 72 hours before the test; cigarette and caffeine (coffee, chocolate, soft drinks) for 24 hours before the test, and fasting for 3 hours before the test. Next, the same subjects underwent the vestibular test a second time, but a different examiner performed it the second time. Vestibular tests were performed by different examiners so that patients were not used to it in the second time. Thus, patients were alert in both tests. Examiners were previously trained so that the first and second tests were exactly the same.

The instructions were the same, except for no interruption of caffeine intake in the second test.

Vectoelectronystagmography was the method used to record ocular movements, applying the triangular placement of electrodes 23. We used the equipment brand Contronic do Brasil version^5^. The assessment of balance comprised the following steps: clinical examination with Frenzel lenses, ocular motricity test and caloric test. We assessed saccadic oculomotor movement, tracking and optokinetic movements, in addition to spontaneous and semi-spontaneous nystagmus. Vestibular assessment included caloric test in temperatures of 30°C and 44°C with water. Data generated for the study were analyzed using the statistical program EPI-INFO 6.04. For the statistical comparison, we applied McNemar test for matched analysis. The level of statistical significance for the study was 5%.

The project and the consent forms were analyzed and approved by the Research Ethics Committee, Federal University of Minas Gerais. All involved subjects agreed to participate in the study and in the dissemination of results, in compliance with Resolution n° 196 of 1996.

## RESULTS

The mean age of participants was 49.5 years, ranging from 21 to 76 years. All subjects were female patients. Mean daily consumption of coffee in the studied population was three cups (Table 1).

**Table 1:** Average consumption of caffeine in patients with complaints of dizziness. N = 19. Sector of Audiology, Hospital das Clínicas, UFMG — Belo Horizonte, 2004.

Number of cups	11g/day	Number of patients (%)
2	100 a 200mgl/day	5 (26,3%)
3	150 a 300mgl/day	6 (31,6%)
4	200 a 400mgl/day	8 (42,1%)

**Graph 1:**
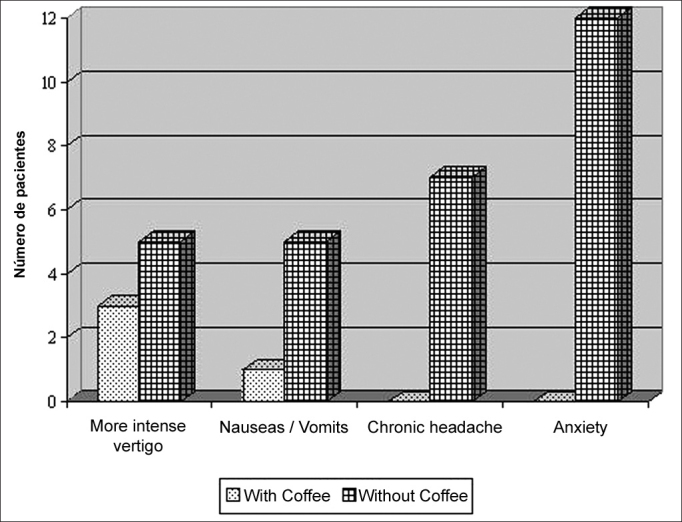
Comparative distribution of complaints in the vestibular test with and without coffee intake. N = 16. Sector of Audiology, Hospital das Clínicas, UFMG — Belo Horizonte, 2004.

The assessment of the most frequent clinical symptoms in relation to vestibular test, with and without coffee intake, are presented in Graph 1. Three patients did not respond to the questionnaire. The highest prevalence of headache and anxiety occurred in patients submitted to the vestibular test with suspension of coffee (p = 0.01).

**Graph 2:**
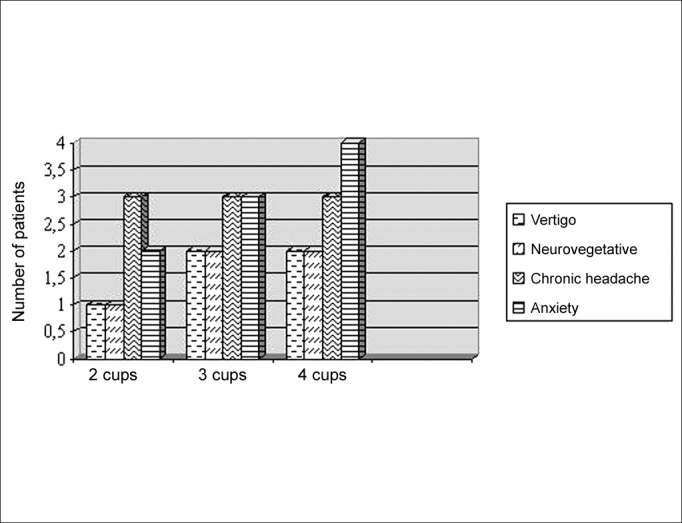
Distribution of symptoms associated with the vestibular test without coffee intake in relation to number of coffee cups taken per day. N = 13. Sector of Audiology, Hospital das Clínicas, UFMG — Belo Horizonte, 2004.

Considering the choice of the patient, 13 (68.4%) preferred to be submitted to the vestibular test with habitual coffee intake. Among the 13 patients, the complaints related with sudden suspension of the drink were anxiety (92.3%), headache (69.3%), nausea and vomiting (38.5%), and more marked vertigo during the test (38.5%). Graph 2 demonstrated that the frequency of withdrawal syndrome has increased with daily dose of caffeine in those patients.

In the result of the vestibular test, no abnormality was observed concerning calibration or measurement of the oculomotor movements with and without coffee suspension. As to caloric test, there was no statistically significant difference between the results found with and without coffee, considering as normal the number of labyrinthic predominance and directional predominance of the nystagmus up to 33%. The matched analysis of data referring to caloric test is demonstrated in Table 2.

**Table 2:** Comparative analysis of results of the calculation of post-caloric nystagmus with and without coffee suspension. N = 17. Sector of Audiology, Hospital das Clínicas, UFMG — Belo Horizonte, 2004.

	Prova Cabrita		
Caloric test	W/out coffee suspension	With coffee suspension	TOTAL
Normal	14	14	28
Abnormal	3	3	6
TOTAL	17	17	34

P value (Mc Nemar) > 0.05.

## DISCUSSION

In agreement with previous studies, dizziness is the most frequent complaint in female gender and it predominates in the 5th and 6th decades of life in both genders^19^^,^^24^.

The preference of the vestibular test performed with habitual coffee intake (68.4%) may be justified by the higher occurrence of anxiety and headache associated with the test when the instruction to interrupt the intake of caffeine was given (p = 0.01).

Caffeine withdrawal syndrome may occur after sudden interruption of intake in those used to moderate or low doses of caffeine^20^^,^^21^. In this study, most participants were used to take daily moderate doses of caffeine (three cups a day)^2^. The complaints of anxiety and headache increased as a result of increased doses of caffeine intake.

Thus, these symptoms present in the test after previous interruption of coffee intake could be manifestations of caffeine withdrawal syndrome, which could negatively interfere in the results of the vestibular test. Anxiety can cause changes to the tracing of vectoelectronystagmography, leading to misdiagnosis of vestibular disease^18^.

The vestibular test is a type of test that may generate some uneasiness in the patient. In the present study, the test with instructions to interrupt coffee intake was the first one to be performed. Thus, anxiety could have been associated with two factors: 1) fear of the exam; 2) caffeine withdrawal syndrome. The highest frequency of headache in the test with previous interruption of caffeine intake, conversely, would not be justified by lack of knowledge about the test. It is interesting to notice that headache is an important symptom in caffeine withdrawal syndrome^12^.

The pharmacological actions of caffeine are well known. In moderate doses, it maintains the subject alert, with greater and more constant intellectual activity, favoring complex association of ideas and reducing the time of reaction to sensorial stimuli^6^. The improvement in the process of vestibular compensation has been demonstrated25., 26., 27.. In high doses, caffeine is toxic to the vestibular system. It may cause impairment of the systems of convergence and accommodation, reduction in saccadic movements and peripheral vestibular dysfunction^28^.

The influence of psychological factors during the performance of a vestibular test has been considered in the quality of the test. To maintain the patient relaxed, and at the same time alert, is essential for the success of the exam. In this study, the result of the vestibular test was similar with and without coffee intake. Therefore, interruption of low and moderate doses of caffeine before the vestibular test should be reassessed, given that in subjects used to take such drinks daily the interruption may trigger undesirable anxiety reactions in subjects that are going to be submitted to the vestibular test.

## CONCLUSION

In the present study, the results of the vestibular test after the intake of moderate doses of coffee were similar to the results with suspension of coffee intake up to 24 hours before the test. Interruption proved to be unnecessary. As to coffee intake instruction before the vestibular test, moderate intake (up to 3 cups/24 hours) may be allowed, with restriction for the last intake up to 6 hours before the test (caffeine half-life).
